# Colonic Intussusception in Adults: A Case Report of an Unusual Cause of Abdominal Pain

**DOI:** 10.7759/cureus.93858

**Published:** 2025-10-05

**Authors:** Luis Fernando Bustamante Sánchez, Erwin Javier Rivera Jiménez, Manuel Israel Vela Martínez, Agustín Castro Segovia, José Luis Cortés Sánchez

**Affiliations:** 1 Surgery, Hospital General Instituto de Seguridad y Servicios Sociales de los Trabajadores del Estado (ISSSTE) "Dr. Francisco Galindo Chávez", Torreón, MEX; 2 Surgery, Hospital General de Zona #21 Instituto Mexicano del Seguro Social (IMSS), San Pedro, MEX; 3 Surgery, Hospital Regional Instituto de Seguridad y Servicios Sociales de los Trabajadores del Estado (ISSSTE) "Lic. Adolfo Lopez Mateos", Mexico City, MEX; 4 Institute of Anatomy, Faculty of Medicine, Otto von Guericke University Magdeburg, Magdeburg, DEU

**Keywords:** causes of intestinal obstruction, colo-rectal cancer, intestinal resection, intussusception in adults, rare cause of abdominal pain

## Abstract

Intussusception is a rare cause of intestinal obstruction in adults, which can present as a complete or partial obstruction. We present the case of a patient who is admitted with abdominal pain in the epigastrium and right hypochondrium, accompanied by weight loss and melanic evacuations. The computed tomography (CT) scan found no adequate passage of oral contrast, and the site of the leading point of intussusception was in the ascending colon. The colonoscopy located a tumor covering approximately 90% of the intestinal lumen. A right hemicolectomy with ileo-transverse lateral-lateral anastomosis was performed, and the pathological report of the surgical specimen reported an infiltrating adenocarcinoma. This case report is significant for the clinical decision-making process it exemplifies.

## Introduction

Intussusception, the telescoping of a proximal segment of the intestine into an adjacent distal segment, is a well-known pathology that occurs predominantly in children. In adults, however, it is an exceptionally rare finding, accounting for only 1% of cases of intestinal obstruction [1]. Unlike its pediatric counterpart, adult intussusception is almost always caused by a pathological lead point, with an underlying malignancy being the etiology in a significant number of cases. Lesions in the small intestine are associated with an underlying neoplasm in approximately 30% of cases, whereas in the colon, this percentage can increase to as high as 65% [2]. The high likelihood of malignancy underscores the critical importance of a prompt and accurate diagnosis in the adult population.

The clinical presentation of adult intussusception is often non-specific and intermittent, presenting a significant diagnostic challenge [3]. Symptoms such as vague abdominal pain, nausea, vomiting, or a palpable mass are common but can mimic other, more prevalent abdominal conditions. This lack of a classic, acute obstructive picture can lead to diagnostic delays, a particularly dangerous situation given the risk of progression to bowel necrosis or perforation. While a high index of clinical suspicion is important, computed tomography (CT) is the diagnostic modality of choice. It is highly effective at visualizing the characteristic "target sign" of intussusception and can often identify the underlying lead point, guiding subsequent therapeutic decisions [2].

Management of adult intussusception is distinctly different from that in children [4]. Due to the high risk of malignancy and potential for compromised vascular supply, surgical intervention is almost always necessary. The choice of surgical approach, whether a minimally invasive laparoscopic procedure or open laparotomy, is a key consideration. While laparoscopy offers the known benefits of reduced postoperative pain and a faster recovery, open conversion may be necessary based on intraoperative findings, such as tumor size or local invasion, or the presence of extensive adhesions [5]. Furthermore, in cases involving a colonic intussusception, it is generally recommended to perform an en bloc resection without reduction to prevent the potential for tumor spillage and to ensure a complete oncologic resection [6].

This case report highlights the complex diagnostic and surgical decision-making procedure for a patient with intussusception caused by a colonic adenocarcinoma. It serves as a valuable clinical reminder of this rare but critical diagnosis, emphasizing the importance of considering intussusception in the differential diagnosis of adult patients with non-specific abdominal symptoms. By detailing the diagnostic challenges and the rationale for our surgical approach, this report aims to provide a useful contribution to the literature and reinforce the management principles of this condition.

## Case presentation

A 68-year-old man was admitted to the emergency department complaining of colic-type abdominal pain localized to the epigastrium and right hypochondrium. The pain had been continuous and postprandial for the past two weeks. The patient also reported a significant weight loss of approximately 13 kg over 6 months and an episode of melanic evacuations two months prior to hospitalization. He denied classic signs of bowel obstruction, such as nausea, vomiting, or obstipation, but did report a notable loss of appetite. The patient reported no significant past medical or family history, and no history of smoking or alcohol abuse. The physical examination revealed cachexia, dehydration, and the presence of bowel sounds. Abdominal tenderness and guarding were negative in the exploration, and no palpable masses were detected. The patient was hemodynamically stable on admission, with a blood pressure of 136/75 mmHg, a respiratory rate of 18, and a slightly increased pulse of 95.

Initial laboratory work revealed anemia with a hemoglobin (Hb) level of 9.9 g/dL. This, combined with the patient's history of weight loss and melanic evacuations, raised a strong suspicion of chronic gastrointestinal blood loss. A lactate level of 1.2 mmol/L (normal range: 0.5-2.2 mmol/L) confirmed that there was no evidence of active tissue ischemia upon admission.

**Figure 1:**
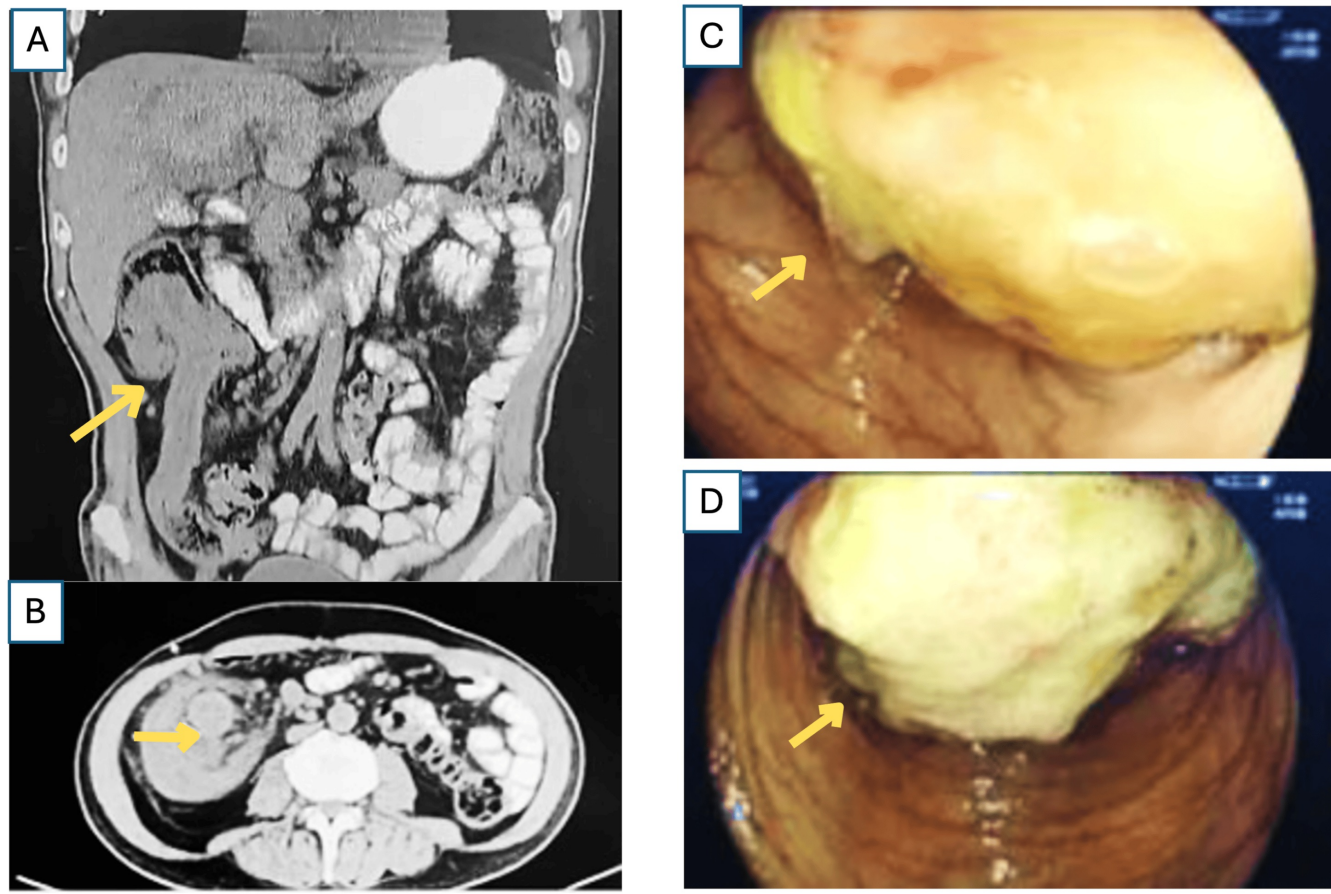
Diagnostic imaging of adult intussusception 1A (coronal) and 1B (axial) Abdominal computed tomography (CT) scan: The axial view of the abdominal CT scan shows the classic "target sign" of an intussusception (indicated by the arrow) in the right colon. The image clearly demonstrates a soft-tissue mass at the center of the invagination, acting as the pathological lead point. Note the presence of surrounding edema and mesenteric fat, which are also consistent with the diagnosis. 1C and 1D Colonoscopy findings: Endoscopic view from the colonoscopy showing a large, fungating tumor with irregular edges (indicated by the arrow) located in the ascending colon. The mass occludes approximately 90% of the lumen, which was identified as the lead point for the intussusception seen on the CT scan.

Given the weight loss and melena, a computed tomography (CT) scan of the abdomen and pelvis was ordered. The scan revealed a classic "target sign" in the right colon, highly suggestive of an intussusception (Figures 1A-1B). The scan also identified a large, soft-tissue mass in the ascending colon acting as the lead point for the intussusception. There was mild upstream bowel dilatation but no evidence of perforation. Following a successful bowel prep, a subsequent colonoscopy was performed to identify a source for the suspicion of chronic gastrointestinal blood loss, which identified a tumor with irregular edges covering 90% of the lumen, located 7 cm from the ascending colon (Figures 1C-1D).

**Figure 2:**
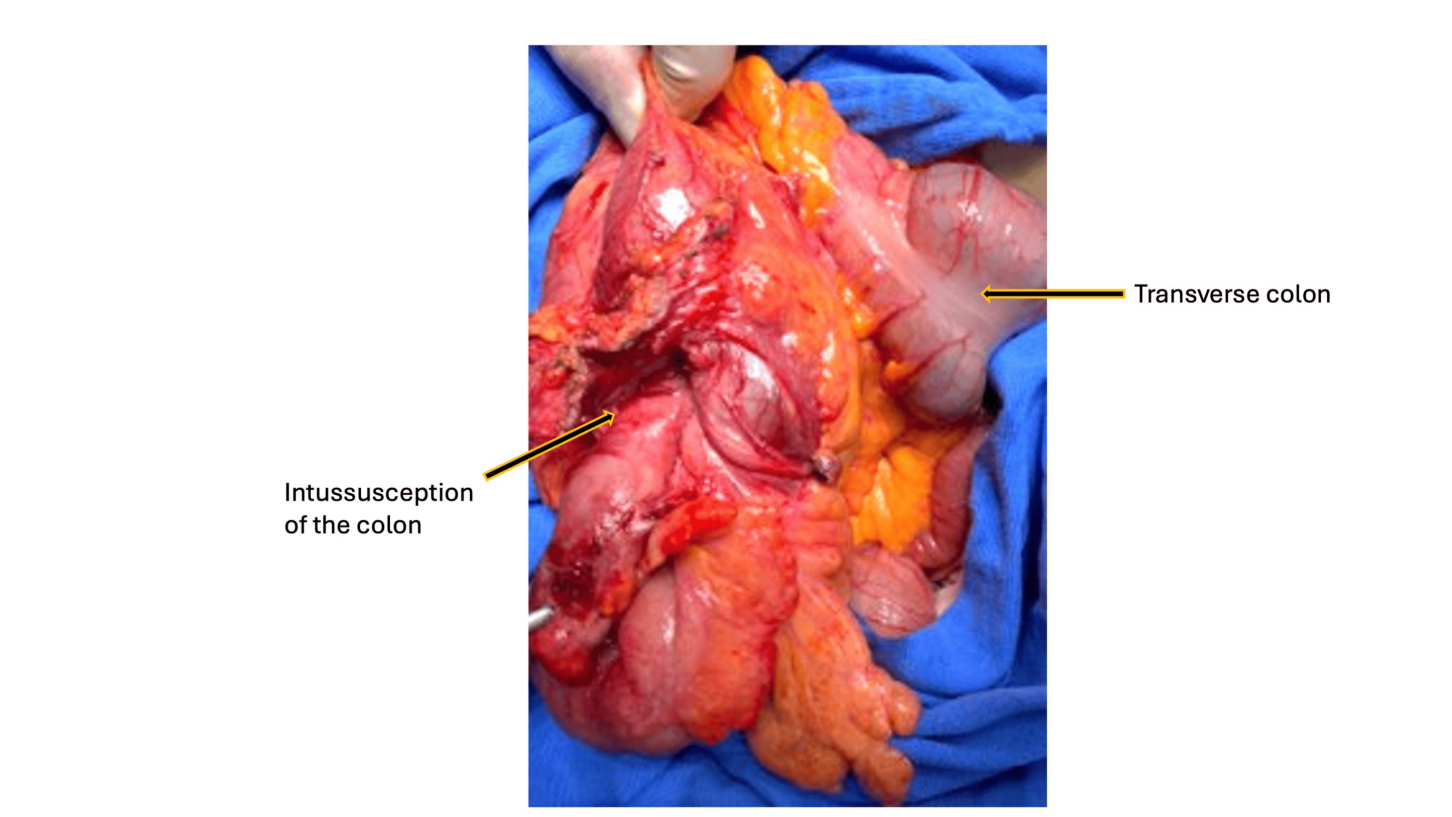
Surgical management Gross image of the resected surgical specimen following an open right hemicolectomy. The specimen includes the terminal ileum, cecum, ascending colon, and a portion of the transverse colon. A well-circumscribed adenocarcinoma measuring approximately 12 x 10 cm served as the lead point. The extensive size of the tumor and the indurated lymph nodes, which are not visible in this view, were the primary reasons for the conversion from a laparoscopic to an open approach to ensure a complete oncological resection.

After a comprehensive diagnostic workup, a semi-elective laparoscopic hemicolectomy was initially attempted. However, due to the tumor's large size (12 x 10 cm) and the presence of significant indurated lymph nodes, the procedure was converted to an open right hemicolectomy. This decision was made to ensure an adequate oncologic resection with clear margins and a complete lymphadenectomy, which would have been technically challenging and potentially unsafe to perform laparoscopically. The surgical specimen, which included the terminal ileum, cecum, ascending colon, and the first third of the transverse colon, was successfully resected (Figure 2). Other significant laboratory findings included elevated tumor markers: carcinoembryonic antigen (CEA) was 21.1 ng/mL (normal range: 0 to 2.5 ng/mL), and CA 19-9 was 124 U/mL (normal range: <37 U/mL).

The patient had an uneventful postoperative course and was discharged on postoperative day 6 in stable condition. A histopathological evaluation of the surgical specimen confirmed an infiltrating adenocarcinoma with a 40% mucinous component. The final staging was pT4a N0 M0, corresponding to a Stage IIB adenocarcinoma of the colon. The patient was subsequently referred to medical oncology for a discussion of adjuvant chemotherapy based on the high-risk features of the stage IIb tumor.

## Discussion

Intussusception is a rare pathology in adults, contrasting with its prevalence in the pediatric population, where up to 90% of cases are idiopathic [1,7]. In adults, an organic cause is identified in the vast majority of cases, making it crucial to investigate for a potential malignancy. The case presented here, caused by an adenocarcinoma, is consistent with this finding, as a neoplastic lead point is responsible for adult intussusception in up to 90% of cases [2]. While intussusception is more common in the small intestine (up to 75% of cases), its occurrence in the colon, as seen in our patient, is relatively rare [1].

The clinical presentation of our patient, characterized by chronic abdominal pain, weight loss, and melanic evacuations, served as a strong indicator of chronic gastrointestinal blood loss and a possible underlying malignancy. The presence of anemia on admission, along with elevated tumor markers (CEA and CA 19-9), significantly heightened the suspicion for a malignant etiology. These findings were key in guiding our diagnostic workup toward definitive imaging and endoscopic evaluation.

The presentation of our patient is a critical teaching point. Despite having an intussusception, there were no signs of a complete bowel obstruction on physical examination or on the abdominal computed tomography (CT) scan. The CT scan showed only mild upstream bowel dilatation, confirming that the intussusception was not completely impassable at the time of presentation. This crucial distinction allowed us to safely perform a preoperative colonoscopy with a preceding bowel preparation. The colonoscopy provided a definitive tissue diagnosis, which not only confirmed the presence of an adenocarcinoma but also enabled a more planned, semi-elective surgical approach rather than an emergency laparotomy. This highlights the importance of a detailed diagnostic approach that integrates clinical suspicion with the appropriate use of imaging and endoscopic evaluation to inform surgical strategy [7].

The choice of imaging modality for a patient with non-specific abdominal pain and suspected intussusception is a critical step. While abdominal ultrasound can be the first-line, non-invasive option in some settings, its diagnostic accuracy is highly operator-dependent and often limited by patient factors. Conversely, a CT scan of the abdomen and pelvis is the imaging modality of choice for suspected intussusception in adults [8]. The CT scan is highly sensitive for detecting the characteristic 'target sign' and the underlying mass, but it cannot definitively determine the histological type of a tumor. Therefore, in this case, the CT scan provided crucial information for surgical planning and staging, but the definitive diagnosis of an adenocarcinoma was only confirmed after the histopathological evaluation of the surgical specimen.

The surgical management of adult intussusception caused by a malignant lead point almost always requires an oncologically sound resection. In this case, the procedure was performed in a semi-elective setting due to the risk of progression to complete obstruction or bowel ischemia. This clinical urgency precluded the possibility of neoadjuvant therapy. The initial attempt at a laparoscopic hemicolectomy, a well-established and accepted practice for colorectal cancer, was converted to an open procedure based on intraoperative findings. The large size of the tumor (12 x 10 cm) and the presence of significant indurated lymph nodes made it clear that a complete oncologic resection with adequate margins and a complete lymphadenectomy could not be performed safely or effectively via a minimally invasive approach. This decision aligns with established surgical principles, which dictate that oncological safety and patient outcome take precedence over the technical approach [9].

The final pathological evaluation of the surgical specimen confirmed an infiltrating adenocarcinoma with clear surgical margins and a total of 15 lymph nodes resected, all of which were negative for metastasis [10]. This detailed pathological report confirmed the final stage as pT4a N0 M0 or Stage IIB colon cancer. The patient had an uneventful postoperative course and was discharged on day 6. As per standard guidelines for high-risk stage IIB colon cancer, the case was presented at a multidisciplinary tumor board meeting, which recommended a course of adjuvant chemotherapy. A structured follow-up plan was initiated, including clinical evaluations and carcinoembryonic antigen (CEA) monitoring every three months, and a surveillance colonoscopy at one year post-resection.

This case report underscores the diagnostic challenge posed by adult intussusception and highlights the importance of a high index of clinical suspicion to ensure timely and appropriate surgical management. It also serves as a reminder that surgical judgment in complex cases, such as the conversion from a laparoscopic to an open procedure, is important for achieving the best possible oncological outcome for the patient.

## Conclusions

Adult intussusception is a rare pathology with a high propensity for a malignant etiology, making it a critical diagnostic and management challenge. While a computed tomography (CT) scan is the definitive diagnostic tool, this case highlights the importance of a high index of clinical suspicion to overcome the diagnostic errors presented by the non-specific, chronic symptoms and the absence of classic obstructive signs.

This case report is significant for the clinical decision-making process it exemplifies. The initial intent to perform a laparoscopic resection was judiciously converted to an open procedure based on the intraoperative findings of a large tumor and extensive regional lymphadenopathy. This decision was a deliberate and necessary step to ensure the highest possible safety. This case serves as a valuable reminder for clinicians to consider intussusception in the differential diagnosis of adult patients with unexplained abdominal pain and to prioritize oncological principles during surgical management.
